# Endovascular treatment for basilar artery occlusion: whether the “weekend effect” affects time metrics and clinical outcomes at a comprehensive stroke center

**DOI:** 10.3389/fneur.2024.1413557

**Published:** 2024-06-27

**Authors:** Jianying Bao, Guangchen Shen, Haibin Shi, Zheng Lin, Sheng Liu

**Affiliations:** ^1^Department of Interventional Radiology, The First Affiliated Hospital of Nanjing Medical University, Nanjing, China; ^2^Department of Radiology, The First Affiliated Hospital of Nanjing Medical University, Nanjing, China; ^3^Department of Nursing, The First Affiliated Hospital of Nanjing Medical University, Nanjing, China

**Keywords:** acute ischemic stroke, basilar artery occlusion, endovascular treatment, weekend effect, comprehensive stroke center

## Abstract

**Objectives:**

This study aimed to evaluate whether the “weekend effect” would affect the time metrics and the prognosis of acute ischemic stroke (AIS) patients who underwent endovascular treatment (EVT) due to basilar artery occlusion (BAO).

**Methods:**

Clinical data of AIS patients who underwent EVT due to BAO between December 2019 and July 2023 were retrospectively analyzed. At the time when the patients were admitted, the study population was divided into the weekdays daytime group and weekends nighttime group. In the subgroup analysis, the study cohort was divided into four groups: the weekdays daytime group, weekdays nighttime group, weekend daytime group, and weekend nighttime group. A good outcome was defined as a modified Rankin Scale score of ≤3 at 90 days after EVT. Time metrics [e.g. onset-to-door time (ODT) and door-to-puncture time (DPT)] and clinical outcomes were compared using appropriate statistical methods.

**Results:**

A total of 111 patients (88 male patients, mean age, 67.7 ± 11.7 years) were included. Of these, 37 patients were treated during weekdays daytime, while 74 patients were treated during nights or weekends. There were no statistically significant differences in ODT (*P* = 0.136), DPT (*P* = 0.931), and also clinical outcomes (*P* = 0.826) between the two groups. Similarly, we found no significant differences in the time metrics and clinical outcomes among the four sub-groups (all *P* > 0.05).

**Conclusion:**

This study did not reveal any influence of the “weekend effect” on the time metrics and clinical outcomes in AIS patients who underwent EVT due to BAO at a comprehensive stroke center.

## Introduction

Basilar artery occlusion (BAO) accounts for 1% of all acute ischemic stroke (AIS) cases and 5% of AIS due to large vessel occlusion (LVO) (1). It is a devastating sub-type of AIS with an extremely poor prognosis. In addition to its proven superiority over the best medical management in both real-world studies and randomized controlled trials, endovascular treatment (EVT) has become an important strategy for treating patients with BAO (1–7). However, as indicated in previous studies, the proportion of patients achieving functional independence at 90 days after EVT is < 40%, even if the occluded artery is successfully recanalized (1, 3). Therefore, clarifying the variables associated with the clinical outcome is crucial for the doctor–patient communication and the establishment of a reasonable treatment strategy.

The “weekend effect”, which was defined as an increased rate of worse outcomes and mortality for hospitalization occurring on weekends or nighttime vs. weekdays, attracts increasing attention (8). It is presumably due to fewer in-hospital personnel and resources during off-hours. Previously, the influence of the “weekend effect” on the clinical outcomes after EVT has been studied sporadically in patients with AIS and mainly in the anterior circulation (8–12). Potts et al. have reported that the door-to-groin times were delayed in patients presenting on the weekends nighttime group compared to weekdays; however, the incidence of symptomatic intracerebral hemorrhage and 90-day good functional outcomes did not differ between the two groups (8). Similarly, Lin et al. found that it took longer during non-working hours than working hours in door-to-image times and door-to-groin puncture times. The change in the National Institute of Health Stroke Scale (NIHSS) scores in 24 h was potentially better in the working-hour group than in the non-working-hour group (9). However, the influence of the working time on the clinical outcomes of patients with BAO after EVT has not been explored until now.

Therefore, the purpose of this study is to explore whether the “weekend effect” existed and its potential influence on the procedural metrics and outcomes of patients with BAO after EVT.

## Methods

### Patient selection

This retrospective study was approved by the institutional review board of our institution. The requirement for written informed consent was waived. We searched for all the patients with posterior circulation AIS who received EVT from December 2019 to July 2023 in our stroke database. Inclusion criteria were as follows: patients (1) whose ages were ≥18 years old, (2) with stroke due to occlusion of BAO, (3) with onset-to-door time (ODT) was < 24 h, (4) with baseline modified Rankin Scale (mRS) scores of 0–2, and (5) on whom EVT was performed. We excluded the patients according to the following criteria: patients (1) with baseline mRS scores of >3, (2) with a combination of anterior circulation stroke, and (3) with incomplete clinical data (e.g., 90-day mRS or time metrics).

### Patients group

Nighttime was defined as the time interval between 6:00 p.m. and 8:00 a.m., while daytime was defined as the remaining hours (12). Weekdays were defined from Monday to Friday, while weekends were defined as Saturday and Sunday (8). Therefore, our study population was divided into four groups according to the time when the patients were admitted into our stroke center: the weekdays daytime group, weekdays nighttime group, weekend daytime group, and weekend nighttime group. After combining the latter three groups, our study cohort was also divided into two groups, namely, the weekdays daytime group and weekends nighttime group.

### Clinical variables

Demographic and clinical data were collected from the database of our stroke center. The following stroke-related risk factors were identified: age, sex, hypertension, hyperlipidemia, diabetes, smoking, and atrial fibrillation. Baseline characteristics, including ODT, door-to-puncture time (DPT), NIHSS score at admission (NIHSS_pre_), NIHSS at 24 h after EVT (NIHSS_24h_), intravenous tissue-type plasminogen activator (IV tPA) performed or not, recanalization status, hemorrhagic transformation (HT) status, and patients outcome, were also collected. Successful recanalization was assessed by using the modified Treatment in Cerebral Infarction (mTICI) scale and defined as mTICI 2b-3 (13). HT was evaluated based on the follow-up CT, where high density persisting in the infarcted area without rapid disappearance was defined as an HT. Good outcomes were defined as an mRS score of ≤ 3 at 90 days after treatment (14).

### Image evaluation and endovascular treatment

One 128-section multi-detector CT scanner (Optima CT 660; GE Healthcare) was used to perform CT scans. Standard non-contrast computed tomography (NCCT) (120 kV, 100–350 auto-mAs, contiguous 5–mm axial sections) and whole-brain volumetric CT perfusion (CTP) scan would be performed for evaluating the patients with AIS. CTP parameters were as follows: four-dimensional adaptive spiral mode, periodic spiral approach, 80 mm in z-coverage, 100 kVp, 200 mAs, rotation time of 0.4 s, 0.984 maximum pitch, and 5 mm thickness. A total of 50 ml of non-ionic iodinated contrast (Iopromide, Ultravist 370, Bayer Schering Pharma) was administered intravenously at 5 ml/sex by using a power injector, followed by 30 ml of saline at the same rate. The total acquisition time was 53 s. Simulated CTA images with a section thickness of 0.625 mm were reconstructed from the peak arterial phase of CTP data for assessing whether an occlusion of BAO existed or not.

EVT was performed using the method reported in a previous study (13). Briefly, EVT was carried out under local anesthesia or conscious sedation. The Solumbra technique was usually performed by using a Solitaire FR device (Medtronic, Irvine, California, USA). If necessary, contact aspiration *via* a 5F or 6F distal access catheter (Penumbra, Alameda, California, USA) was performed. After each intervention, angiography was performed to evaluate blood flow restoration. For patients with residual stenosis but acceptable reperfusion, antiplatelet and/or statin medicines would be suggested. For patients with residual stenosis and *insitu* thrombosis, balloon angioplasty and/or stent implantation could be considered according to the operator′s experience. Intra-arterial thrombolysis or tirofiban administration would also be used as rescue therapies.

### Statistical analysis

Statistical analyses were performed using SPSS version 26.0 (IBM Corporation). Continuous variables were presented as mean ± standard deviation (SD) or median with interquartile range (IQR) depending on the distribution of variables. The normality of the distributions was evaluated using Shapiro–Wilk tests. Categorical variables were presented as numbers and percentages. Comparisons of continuous variables between two groups were performed using independent sample *t*-tests if normally distributed or using the Mann–Whitney U test if not normally distributed. Comparisons of continuous variables among four groups were performed using one-way analysis of variance analyses, followed by multiple comparisons using least-significant difference or Tamhans, as appropriate, to identify where the differences lay. Comparisons of categorical variables were performed using the Chi-square test or Fisher's exact test. A two-sided *P-*value of < 0.05 was considered to be statistically significant.

## Results

Patients' characteristics are detailed in Table 1. Of the 111 patients (88 male patients, mean age, 67.7 ± 11.7 years old) finally included, the mean ODT and DPT were 433.2 ± 332.2 min and 126.3 ± 214.9 min, respectively. A total of 32 patients (28.8%) were administered IV rt-PA before EVT. Successful recanalization was achieved in 94 (84.7%) patients, and 33 (29.7%) patients achieved good outcomes at 90 days after EVT.

**Table 1:** Patient characteristics.

**Variables**	**Number**
Male, *n* (%)	88 (79.3%)
Mean age, y (SD)	67.7 (11.7)
Mean ODT, min (SD)	433.2 (332.2)
Mean DPT, min (SD)	126.3 (214.9)
Mean NIHSS_pre_ (SD)	21.5 (12.8)
Mean NIHSS_24h_ (SD)	19.5 (14.7)
Hypertension, *n* (%)	77 (69.4%)
Hyperlipidemia, *n* (%)	1 (1.8%)
Diabetes, *n* (%)	33 (29.7%)
Smoker, *n* (%)	14 (12.6%)
Atrial fibrillation, *n* (%)	19 (17.1%)
IV tPA, *n* (%)	32 (28.8%)
Successful recanalization, *n* (%)	94 (84.7%)
Hemorrhagic transformation, *n* (%)	26 (23.4%)
Good outcome, *n* (%)	33 (29.7%)

SD, standard deviation; ODT, onset to door time; DPT, door to puncture time; NIHSSpre, National Institutes of Health Stroke Scale scores at admission; NIHSS_24h_, NIHSS at 24 hours after treatment; IV, intravenous; and tPA, tissue-type plasminogen activator. *n* indicates the patients' number. Continuous variables were expressed as mean (standard deviation) due to normal distribution, and categorical variables were expressed as numbers (percentages).

A total of 37 patients were treated during weekdays daytime, and 74 patients were treated during nights or weekends. Table 2 compares the baseline demographics, risk factors, and presenting characteristics between the two groups. There were no statistically significant differences in any variables between the two groups (all *P* > 0.05), other than that a higher proportion of patients underwent administration of IV tPA in the weekend nighttime group (*P* = 0.046).

**Table 2:** Comparisons between the weekdays daytime and weekends nighttime groups.

**Variables**	**Weekdays daytime (*n* = 37)**	**Weekends nighttime (*n* = 74)**	***P***
Male, *n* (%)	31 (83.8%)	54 (77.0%)	0.466
Mean age, *y* (SD)	69.4 ± 12.0	66.8 ± 11.6	0.278
Mean ODT, min (SD)	499.8 ± 319.7	399.8 ± 335.4	0.136
Mean DPT, min (SD)	128.8 ± 246.5	125.0 ± 198.9	0.931
Mean NIHSS_pre_ (SD)	20.0 ± 13.3	22.3 ± 12.6	0.385
Mean NIHSS_24h_ (SD)	18.1 ± 15.1	20.3 ± 14.5	0.461
Hypertension, *n* (%)	27 (73.0%)	50 (67.6%)	0.664
Hyperlipidemia, *n* (%)	0 (0.0%)	2 (2.7%)	0.552
Diabetes, *n* (%)	8 (21.6%)	25 (33.8%)	0.271
Smoker, *n* (%)	7 (18.9%)	7 (9.5%)	0.224
Atrial fibrillation, *n* (%)	5 (13.5%)	14 (18.9%)	0.597
IV tPA, *n* (%)	6 (16.2%)	26 (35.1%)	0.046
Successful recanalization, *n* (%)	30 (81.1%)	64 (86.5%)	0.577
Hemorrhagic transformation, *n* (%)	8 (21.6%)	18 (24.3%)	0.816
Good outcome, *n* (%)	12 (32.4%)	21 (28.4%)	0.826

SD, standard deviation; ODT, onset to door time; DPT, door to puncture time; NIHSSpre, National Institutes of Health Stroke Scale scores at admission; NIHSS_24h_, NIHSS at 24 hours after treatment; IV, intravenous; and tPA, tissue-type plasminogen activator. *n* indicates the patients' number. Categorical variables were expressed as numbers (percentages). Continuous variables were presented as mean (standard deviation) due to normal distribution.

**Figure 1:**
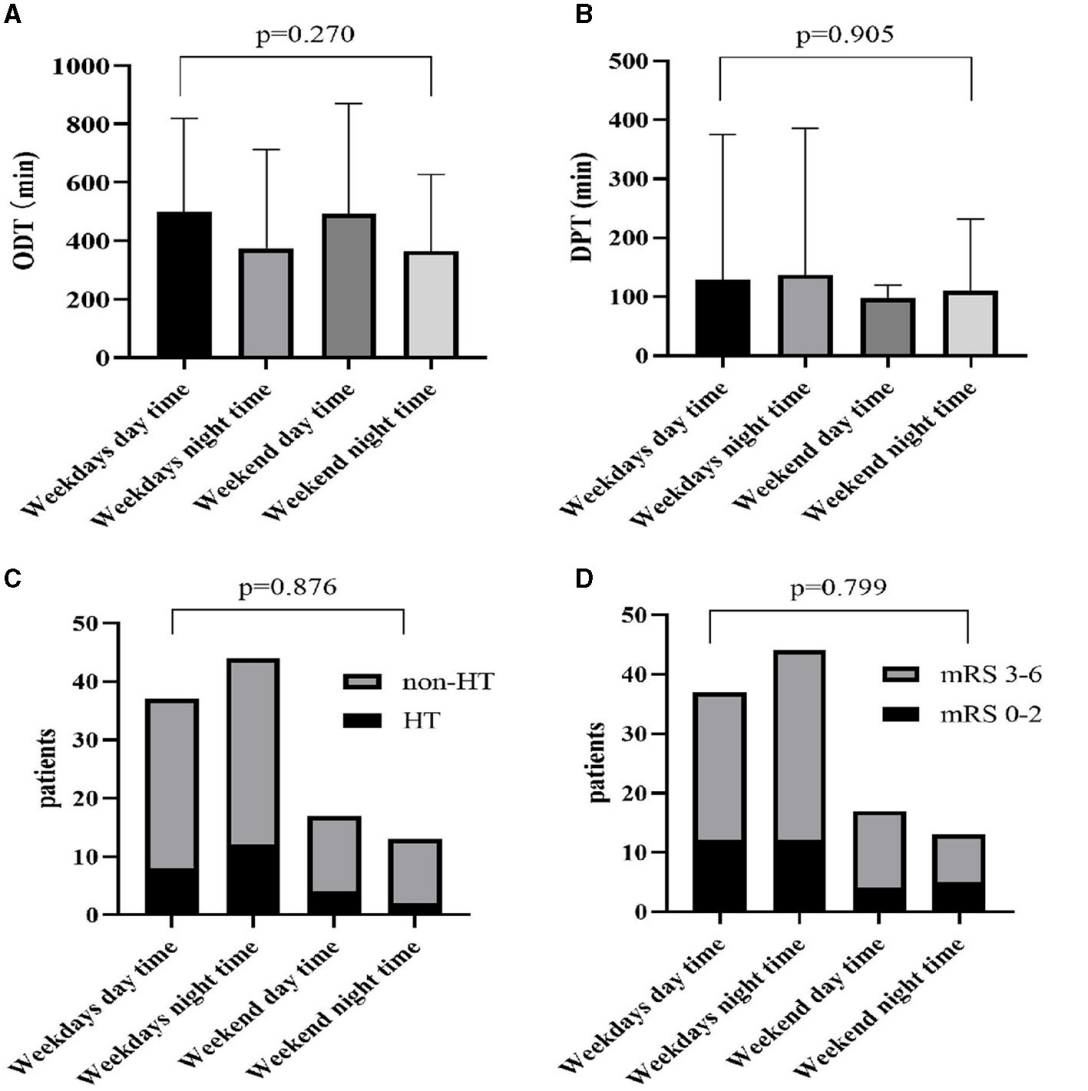
Differences of ODT **(A)**, DPT **(B)**, HT **(C)**, and mRS **(D)** among four groups. ODT, onset-to-door time; DPT, door-to-puncture time; HT, hemorrhagic transformation; mRS, modified Rankin Scale.

To further investigate differences in the time metrics and outcomes, we divided the cohort into four subgroups, namely, the weekdays daytime group, weekdays nighttime group, weekend daytime group, and weekend nighttime group (Table 3). Similarly, we did not find any significant differences in the demographics, risk factors, time metrics, and patient outcomes among the four subgroups (all *P* > 0.05). The differences between ODT, DPT, HT, and mRS among the four groups are shown in Figure 1.

**Table 3:** Comparisons among four subgroups.

**Variables**	**Weekdays daytime (*n* = 37)**	**Weekdays nighttime (*n* = 44)**	**Weekend daytime (*n* = 17)**	**Weekend nighttime (*n* = 13)**	***P1***	***P2***	***P3***	***P4***	***P5***	***P6***	***P***
Male, *n* (%)	31 (83.8%)	35 (79.5%)	13 (76.5%)	9 (69.2%)	0.776	0.707	0.420	>0.999	0.466	0.698	0.696
Mean age, *y* (SD)	69.4 ± 12.0	66.5 ± 12.1	68.7 ± 9.4	65.5 ± 12.9	0.267	0.853	0.311	0.498	0.801	0.459	0.610
Mean ODT, min (SD)	499.8 ± 319.7	374.5 ± 338.2	492.2 ± 377.8	365.2 ± 261.5	0.092	0.938	0.210	0.215	0.929	0.299	0.270
Mean DPT, min (SD)	128.8 ± 246.5	140.4 ± 250.5	97.2 ± 22.2	110.3 ± 122.1	0.811	0.621	0.792	0.489	0.662	0.871	0.905
Mean NIHSS_pre_ (SD)	20.0 ± 13.3	22.8 ± 12.0	22.0 ± 14.0	20.6 ± 13.8	0.328	0.599	0.883	0.821	0.587	0.772	0.788
Mean NIHSS_24h_ (SD)	18.1 ± 15.1	21.5 ± 14.3	21.8 ± 15.1	13.9 ± 14.0	0.291	0.385	0.380	0.947	0.102	0.146	0.323
Hypertension, *n* (%)	27 (73.0%)	30 (68.2%)	13 (76.5%)	7 (53.8%)	0.807	>0.999	0.301	0.755	0.509	0.255	0.579
Hyperlipidemia, *n* (%)	0 (0.0%)	0 (0.0%)	1 (5.9%)	1 (7.7%)	>0.999	0.315	0.260	0.279	0.228	>0.999	0.071
Diabetes, *n* (%)	8 (21.6%)	14 (31.8%)	8 (47.1%)	3 (23.1%)	0.329	0.106	>0.999	0.373	0.734	0.259	0.279
Smoker, *n* (%)	7 (18.9%)	4 (9.1%)	1 (5.9%)	2 (15.4%)	0.329	0.411	>0.999	>0.999	0.611	0.565	0.510
Atrial fibrillation, *n* (%)	5 (13.5%)	6 (13.6%)	4 (23.5%)	4 (30.8%)	>0.999	0.439	0.214	0.444	0.213	0.698	0.377
IV tPA, *n* (%)	6 (16.2%)	17 (38.6%)	5 (29.4%)	4 (30.8%)	0.029	0.293	0.420	0.565	0.748	>0.999	0.162
Successful recanalization, *n* (%)	30 (81.1%)	38 (86.4%)	14 (812.4%)	12 (92.3%)	0.577	>0.999	0.662	0.699	>0.999	0.613	0.828
Hemorrhagic transformation, *n* (%)	8 (21.6%)	12 (27.3%)	4 (23.5%)	2 (15.4%)	0.613	>0.999	>0.999	>0.999	0.484	0.672	0.876
Good outcome, *n* (%)	12 (32.4%)	12 (27.3%)	4 (23.5%)	5 (38.5%)	0.634	0.749	0.741	>0.999	0.499	0.443	0.799

SD indicates standard deviation; ODT, onset to door time; DPT, door to puncture time; NIHSSpre, National Institutes of Health Stroke Scale scores at admission; NIHSS_24h_, NIHSS at 24 h after treatment; IV, intravenous; and tPA, tissue-type plasminogen activator. *n* indicates the patients' number. Categorical variables were expressed as numbers (%). Continuous variables were presented as mean (standard deviation) due to normal distribution. *P* value: comparison among four groups. *P1* value: comparison between weekdays daytime vs. weekdays nighttime. *P2* value: comparison between weekdays daytime vs. weekend daytime. *P3* value: comparison between weekdays daytime vs weekend nighttime. *P4* value: comparison between weekdays nighttime vs. weekend daytime. *P5* value: comparison between weekdays nighttime vs. weekend nighttime. *P6* value: comparison between weekend daytime vs. weekend nighttime.

## Discussion

The “Weekend effect,” which was termed as a poor outcome due to fewer in-hospital personnel and resources during off-hours, had been focused on for several years. Previously, several studies had tried to explore the potential influence of the “weekend effect” on the clinical outcomes after EVT in patients with AIS (8–12); however, these studies mainly focused on stroke due to anterior circulation LVO. The present study first focused on the potential influence of working time on the clinical outcomes of patients with BAO after EVT and found that there were no statistically significant differences in ODT, DPT, and also clinical outcomes among groups with different working times. Our study indicated that the “weekend effect” might not exist in patients with AIS who underwent EVT due to BAO at a comprehensive stroke center.

Previously, several studies had investigated the potential existence and influence of the “weekend effect” of EVT on patients with AIS, especially those due to anterior circulation LVO (8–12). Mpotsaris et al. reported that the patients admitted during nighttime and weekends showed statistically prolonged door-to-reperfusion times; however, it did not affect the rate of revascularization and favorable outcome (12). This main conclusion was also supported by similar studies by Potts et al. (8), Lin et al. (9), and Omura et al. (10).. They explained that the outpatient clinics did not provide services, which led to the patients being crowded into the emergency department during non-working hours, and subsequently slightly increased door-to-image times. In addition to that, the team in the comprehensive stroke center included several specialties (e.g., physician, stroke neurology, neurointervention surgery, radiologists, and nurses), and some of them were on-call from home during non-working hours (9). Prolonged door-to-reperfusion times might be due to waiting for some specialties on duty during non-working hours. However, further analysis did not find a significant influence of increased time intervals on the functional outcome. This might be due to the wide application of perfusion imaging for patient selection. An accurate assessment of the “tissue-window” might offset the influence of a prolonged “time-window” on the functional outcome (15).

Recently, numerous case series and trials have reported the efficacy of EVT in patients with AIS due to BAO (1–7, 16). As so many positive results had been reported, we could expect that the number of EVTs for treating patients with BAO would significantly increase. However, the impact of the “weekend effect” on time metrics and clinical outcomes has not been fully studied to date. Our study focused on this topic for the first time, and we found that there were no statistically significant differences in ODT, DPT, and clinical outcomes between the weekdays daytime group and weekends nighttime group and also in further subgroup analysis. It was not surprising that the difference in clinical outcomes was not significant, especially because the treatment time window of posterior circulation stroke was longer than that of anterior circulation stroke (17). Nevertheless, we also did not observe a prolonged time metrics in the weekends nighttime group. In the author's opinion, it might be due to that center being a comprehensive stroke center in the academic setting. Our group included emergency physicians, radiologists, neurointerventional team, and had 24/7 availability of all personnel involved in the emergency treatment of AIS. Except for nurses and technicians in the neurointerventional team, all the other members in our group were “on-call” in the hospital and not “in-house.” Because our hospital was an academic unit, the in-hospital residents and fellows could expedite the treatment process.

There were some limitations to our study. The first limitation is that this was a retrospective study conducted in a single center with a relatively small sample size; therefore, the selection bias was inevitable. The second limitation is our results were specific to the EVT situation (mostly high-volume stroke centers) and might differ from other centers with different organizations of acute stroke therapy.

In conclusion, based on a retrospective cohort of patients with AIS due to BAO from a comprehensive stroke center, we did not observe any differences in time metrics and clinical outcomes after EVT between the weekdays daytime group and weekend nighttime group. The “weekend effect” might not exist in patients with BAO who underwent EVT in a well-organized comprehensive stroke center. In future, further multiple-center study with larger sample sizes was warranted to confirm our results.

## Data availability statement

The original contributions presented in the study are included in the article/supplementary material, further inquiries can be directed to the corresponding authors.

## Ethics statement

The studies involving humans were approved by the First Affiliated Hospital of Nanjing Medical University. The studies were conducted in accordance with the local legislation and institutional requirements. The participants provided their written informed consent to participate in this study.

## Author contributions

JB: Writing – original draft. GS: Data curation, Writing – review & editing. HS: Writing – review & editing, Funding acquisition, Supervision. ZL: Writing – review & editing. SL: Conceptualization, Methodology, Supervision, Writing – review & editing.

## References

[1] Tao C Qureshi AI Yin Y Li J Li R Xu P. Endovascular treatment versus best medical management in acute basilar artery occlusion strokes: results from the attention multicenter registry. Circulation. (2022) 146:6–17. 10.1161/CIRCULATIONAHA.121.058544 35656816

[2] Huo X Lv X. Mechanical thrombectomy. In: Lv X Wang G Wang J, editor Craniospinal Vascular Diseases and Endovascular Neurosurgery. NY, USA: Nova Science (2021). p. 455–480.

[3] Jovin TG Li C Wu L Wu C Chen J Jiang C. Trial of thrombectomy 6 to 24 hours after stroke due to basilar-artery occlusion. N Engl J Med. (2022) 387:1373–84. 10.1056/NEJMoa2207576 36239645

[4] Tao C Nogueira RG Zhu Y Sun J Han H Yuan G. Trial of endovascular treatment of acute basilar-artery occlusion. N Engl J Med. (2022) 387:1361–72. 10.1056/NEJMoa2206317 36239644

[5] Langezaal LCM van der Hoeven EJRJ. Mont'Alverne FJA de Carvalho JJF Lima FO Dippel DWJ. Endovascular therapy for stroke due to basilar-artery occlusion. N Engl J Med. (2021) 384:1910–20. 34010530 10.1056/NEJMoa2030297

[6] Zi W Qiu Z Wu D Li F Liu H Liu W. Assessment of endovascular treatment for acute basilar artery occlusion via a nationwide prospective registry. JAMA Neurol. (2020) 77:561–573. 10.1001/jamaneurol.2020.0156 32080711 PMC7042866

[7] Liu X Dai Q Ye R Zi W Liu Y Wang H. Endovascular treatment versus standard medical treatment for vertebrobasilar artery occlusion (BEST): an open-label, randomised controlled trial. Lancet Neurol. (2020) 19:115–22. 10.1016/S1474-4422(19)30395-3 31831388

[8] Potts MB Abdalla RN Golnari P Sukumaran M Palmer AH Hurley MC. Analysis of Mechanical Thrombectomy for Acute Ischemic Stroke on Nights and Weekends Versus Weekdays at Comprehensive Stroke Centers. J Stroke Cerebrovasc Dis. (2021) 30:105632. 10.1016/j.jstrokecerebrovasdis.2021.105632 33517033

[9] Lin CW Huang HY Guo JH Chen WL Shih HM Chu HT. Does weekends effect exist in Asia? Analysis of endovascular thrombectomy for acute ischemic stroke in a medical center. Curr Neurovasc Res. (2022) 19:225–31. 10.2174/1567202619666220727094020 35894472 PMC9900696

[10] Omura N Kakita H Fukuo Y Shimizu F. Differences in mechanical thrombectomy for acute ischemic stroke on weekdays versus nights/weekends in a Japanese primary stroke core center. J Cerebrovasc Endovasc Neurosurg. (2023) 25:297–305. 10.7461/jcen.2023.E2023.01.006 37433465 PMC10555624

[11] Herm J Schlemm L Siebert E Bohner G Alegiani AC Petzold GC. How do treatment times impact on functional outcome in stroke patients undergoing thrombectomy in Germany? Results from the German stroke registry. Int J Stroke. (2021) 16:953–61. 10.1177/1747493020985260 33472575

[12] Mpotsaris A Kowoll A Weber W Kabbasch C Weber A Behme D. Endovascular stroke therapy at nighttime and on weekends-as fast and effective as during normal business hours? J Vasc Interv Neurol. (2015) 8:39–45. 25825631 PMC4367806

[13] Liu XL Hang Y Cao Y Jia Z Zhao LB Shi HB. Tmax profile in computed tomography perfusion-based RAPID software maps influences outcome after mechanical thrombectomy in patients with basilar artery occlusion. J Neurointerv Surg. (2023) 15:639–43. 10.1136/neurintsurg-2021-018557 35580984

[14] Shen GC Hang Y Ma G Lu SS Wang C Shi HB. Prognostic value of multiphase CT angiography: estimated infarct core volume in the patients with acute ischaemic stroke after mechanical thrombectomy. Clin Radiol. (2023) 78:e815–22. 10.1016/j.crad.2023.07.015 37607843

[15] Wang D Wang Y. Tissue window, not the time window, will guide acute stroke treatment. Stroke Vasc Neurol. (2019) 4:1–2. 10.1136/svn-2018-000211 31105971 PMC6475083

[16] Nguyen TN Fisher M Schonewille WJ. Evolution of endovascular therapy trials for basilar artery occlusion. J Cereb Blood Flow Metab. (2023) 43:2005–7. 10.1177/0271678X231187174 37409675 PMC10676134

[17] Powers WJ Rabinstein AA Ackerson T Adeoye OM Bambakidis NC Becker K. 2018 Guidelines for the early management of patients with acute ischemic stroke: a guideline for healthcare professionals from the American heart association/American stroke association. Stroke. (2018) 49:e46–e110. 10.1161/STR.0000000000000158 29367334

